# Hardware-Based Hopfield Neuromorphic Computing for Fall Detection

**DOI:** 10.3390/s20247226

**Published:** 2020-12-17

**Authors:** Zheqi Yu, Adnan Zahid, Shuja Ansari, Hasan Abbas, Amir M. Abdulghani, Hadi Heidari, Muhammad A. Imran, Qammer H. Abbasi

**Affiliations:** 1 James Watt School of Engineering, University of Glasgow, Glasgow G12 8QQ, UK; a.zahid.1@research.gla.ac.uk (A.Z.); shuja.ansari@glasgow.ac.uk (S.A.); hasan.abbas@glasgow.ac.uk (H.A.); hadi.heidari@glasgow.ac.uk (H.H.); muhammad.imran@glasgow.ac.uk (M.A.I.); Qammer.Abbasi@glasgow.ac.uk (Q.H.A.); 2 School of Engineering and Physical Sciences, Heriot Watt University, Edinburgh EH14 4AS, UK; 3 Department of Electrical and Computer Engineering, Sultan Qaboos University, Muscat 123, Oman; amirm@squ.edu.om

**Keywords:** neuro-inspired model, neural networks, signal processing, artificial intelligence

## Abstract

With the popularity of smart wearable systems, sensor signal processing poses more challenges to machine learning in embedded scenarios. For example, traditional machine-learning methods for data classification, especially in real time, are computationally intensive. The deployment of Artificial Intelligence algorithms on embedded hardware for fast data classification and accurate fall detection poses a huge challenge in achieving power-efficient embedded systems. Therefore, by exploiting the associative memory feature of Hopfield Neural Network, a hardware module has been designed to simulate the Neural Network algorithm which uses sensor data integration and data classification for recognizing the fall. By adopting the Hebbian learning method for training neural networks, weights of human activity features are obtained and implemented/embedded into the hardware design. Here, the neural network weight of fall activity is achieved through data preprocessing, and then the weight is mapped to the amplification factor setting in the hardware. The designs are checked with validation scenarios, and the experiment is completed with a Hopfield neural network in the analog module. Through simulations, the classification accuracy of the fall data reached 88.9% which compares well with some other results achieved by the software-based machine-learning algorithms, which verify the feasibility of our hardware design. The designed system performs the complex signal calculations of the hardware’s feedback signal, replacing the software-based method. A straightforward circuit design is used to meet the weight setting from the Hopfield neural network, which is maximizing the reusability and flexibility of the circuit design.

## 1. Introduction

With the ever-improving living conditions, sensors-based healthcare has been widely adopted. Embedded systems are used as monitoring tools for well-being or preventive purposes. Such systems are composed of non-invasive and wearable sensors with processors [1]. Sensors acquire a variety of physiological signals and can be used for life-saving implants, medical treatments, and long-term health monitoring of disabled or elderly persons [2]. For example, real-time, reliable and accurate monitoring results provided by the sensor system are used for fall recognition [3], blood glucose monitoring [4] and asthma tracking [5].

Presently, machine learning is being widely used for various applications in diverse fields and health care is no exception. It can be used to improve the accuracy of monitoring and detection, which is a popular method in the wearable field [6]. However, the combination of machine learning and embedded devices still has a technological divide. Primarily, it is a huge challenge to design low-power and real-time healthcare artificial intelligence (AI) systems. For instance, an embedded system is not capable of running an AI algorithm of neural network for low-power and real-time processing of multi-sensor signal [7], such as data fusion of magnetic, acceleration and RF/Radar sensor signal processing for activity detection. Furthermore, if real-time cloud solutions were used to collect and process the wearable device signals on the host computer, it would use more data and become inconvenient and a risk for customers [8].

The problem is that the neural network algorithm requires a large number of differential equations to be solved, to compute gradient descent, such as mean square error and regularization calculations. The instruction set and architecture of a microprocessor constrain these software-based calculations on a general-purpose processor, and it requires many consumption thread resources to complete. In this respect, hardware-based neuromorphic computing systems present a more efficient AI technology, that has been proposed by researchers and scientists at various levels. It aims to stimulate neurons using specialized hardware that uses the discrete and sparse nature of neuron pulse behavior to achieve desired results.

Neuromorphic computing hardware refers to a hardware system that supports the scale of simulated neural network models and the speed of neural computing. Its initial hardware implementation includes Field Programmable Gate Array (FPGA) [9], Neuromorphic Chip (Based on ASIC) [10] and Digital Signal Processor (DSP) [11]. The core of hardware implementation research is the construction of neural devices, which can be of electronic [12], optical [13] and biological [14] nature. In the research of neuromorphic computing, for neural network algorithms to be effectively applied in applications, the neural network implementation technology request to support the neuromorphic computing of scalable network architecture. Meanwhile, the system shall minimize the cycle time of the neuromorphic computing process to match real-time processing. However, the existing system based on the software environment of various AI algorithms is difficult to support the large-scale and running time of the neural network model, which cannot meet the application requirements. Therefore, the development of hardware-implemented neural network computers is imperative to replace the software environment and achieve hardware acceleration.

Traditional neuromorphic computing of Hopfield neural network algorithms has been applied to data classification that relies on associative memory model. Data is processed directly by storing and memorizing standard data models. For example, Rong and Junfei [15] tried to use the network for water quality detection. They designed a memory template by selecting the water quality parameters for data preprocessing, and then passed the actual data back into the network. They used the binary values 1 and −1 to indicate the water quality monitoring and finally output the feedback matrix to match the classes of the water quality information in the memory models. Cantini et al. [16] used the memory retrieval of the Hopfield neural network to describe gene expression patterns and analyze the transcriptome data. They used discrete values to represent the signature genes in the sample and assigned corresponding neurons to them, where the network used a different gene to mark the nodes. With the network convergence, the model finally evolved to classify the genetic data. In another study, Ray and Majumder [17] used the Hopfield neural network to perform feature matching on a two-dimensional array. By comparing the features of the test scene and the object model, they used the neuron to output the probability to achieve the data classification. López et al. [18] implemented the Hopfield neural network on embedded systems such as the Arduino UNO, Tiva-C and BeagleBone development boards, and achieved fast execution times that even performed machine-learning algorithms. Furthermore, Boriskov [19] proposed Hopfield algorithm uses rate coding to expand the capabilities of neuromorphic engineering by hardware design that uses the thresholds of zero crossings of output voltages of neurons where spiking frequencies of oscillators are controlled either by supply currents or by variable resistances. However, the main disadvantages of these hardware implementations are their limited processing and storage capacity, therefore, such systems can only be used when there is scarce data [18], and request special hardware component design (such as leaky integrated-and-fire (LIF) RC oscillators) [19] to achieve neuron function. It greatly limits scalability and extensibility of hardware-based neuromorphic computing implementation.

This paper focuses on the use of neuromorphic computing algorithms on hardware circuits, so that low-power and real-time systems particularly for health care applications can be implemented. It uses hardware circuit features for algorithm calculations, to reduce power consumption due to the complexity of the computing architecture. The algorithm adopts the integrated structure of storage and computing like human brain processing. It is different from the von Neumann architecture where storage and computing are separated, and can process data more efficiently as a neuron model. The neuromorphic computing can process data in real time that does not require data interaction between multiple modules during the calculation. In the Al field, its conditions in terms of flexible architecture, modular design, and non-declining computing efficiency are rather good, and development potential is very significant [20]. Meanwhile, the neuromorphic computing of the feasibility of the design way of hardware, which to take advantage of low power consumption and real-time processing [21].

The paper is structured as follows. Section 2 introduces the neuromorphic computing with data classification of the Hopfield neural network. Section 3 shows the details of hardware architecture. In Section 4, fall recognition results and comparison of the application with other AI methods is presented, which discusses in detail the hardware-based neuromorphic computing and software-based machine-learning method. Section 5 summarizes the entire hardware implementation of the Hopfield neuromorphic computing algorithm and outlines the potential future direction.

## 2. Neuromorphic Computing Implementation

Traditional human fall recognition techniques mostly rely on the microprogrammed control unit (MCU)-based embedded systems that only sense but process human activity. However, such an approach has an inherent disadvantage in terms of the speed with which any recognition can be detected. Therefore, it is difficult to support the complex calculations any machine-learning algorithm requires. Moreover, most embedded systems are limited to low power, and hence sensor data cannot be processed in real time, something which is critical in designing prediction-based systems.

**Figure 1:**
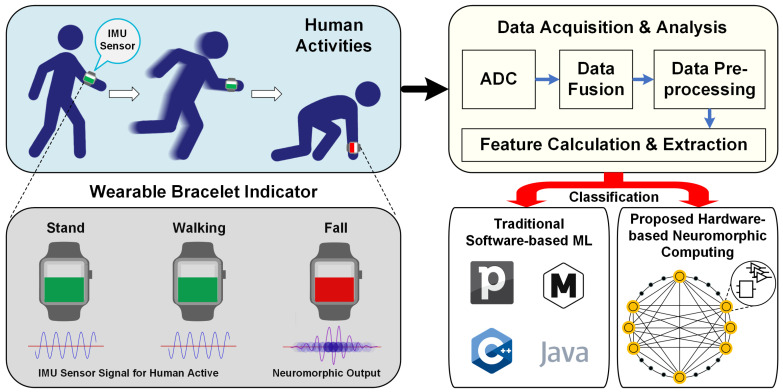
System Workflow from human activities to fall detection.

In this paper, we propose a system that first collects data in real time from a IMU sensor the related to human activities, and then pre-processes this sensor data by posture calculation to estimate the fall feature during human movement. Finally, the data is passed to a hardware-based neural network, which replaces the software-based machine-learning method to perform the recognition calculations. The proposed hardware-based neural network has lower power consumption and real-time processing speed, and thus, meets application requirements for human activity recognition scenarios. Here, we design a hardware circuit that implements a Hopfield neural network algorithm. Through our system, the human fall activity can be processed on analog circuits. In this paper, the entire process from the beginning of the IMU sensor data collection to the final recognition result on the circuits has been built and verified in a simulation environment. The experiment completed the Hopfield neural network in the analog module by Cadence PSpice 17.4, and through the co-simulation of MATLAB Simulink 2020B. As shown in Figure 1, the sensor is worn on the wrist, and the real-time signal can be collected at a sampling rate of 50 Hz, which is then passed to the front-end circuit for data preprocessing. The next step involves processing by the neural network algorithm, hardware done on the system hardware. In Section 3, we describe in detail the working principle of the algorithm, specifically, how neuromorphic calculations are generated based on Hopfield neural network. We further illustrate how to design and build the analog circuit which can further enable the production of useful feedback signals.

### Hopfield Neural Network Algorithm and Training

The Hopfield neural network is a fully connected network proposed by J. Hopfield in 1982 [22] that can be used as an associative memory. It is a recurrent neural network, which has feedback connections from input to output. All neurons are the same structure and connected. Each neuron receives feedback information from other neurons through connection weights, and the signal can transfer in both positive and negative directions. Such a design allows the output of neurons to be controlled by all other neurons, so that each neuron can interact with each other.

Hopfield network can be divided into discrete Hopfield neural network (DHNN) and continuous Hopfield neural network (CHNN) [23]. Continuous Hopfield neural network is mainly used for optimization calculation, and discrete Hopfield neural network is primarily used for associative memory. Among them, the neuron variable function of discrete Hopfield network is symbolic. The node states of the network take the binarized +1 and −1. Hopfield neural network is derived from a nonlinear dynamical system, and DHNN can be described by a set of nonlinear difference equations [24], while differential equations usually describe to the CHNN [25]. Compared to other machine-learning algorithms, the Hopfield neural network is more straightforward and less dependent on the data.

Discrete Hopfield neural network (DHNN) is an essential type of Hopfield neural network, where both the input and output are binarized. The synaptic weight between neuron *i* and neuron *j* is *W*${}_{ij}$ [26], so for a Hopfield neural network with N neurons, the weight matrix size is *N* × *N*, and its unique associative memory of DHNN is through a series of iterative processes until the system is stable. Meanwhile, neurons in the network are connected symmetrically, i.e., *W*${}_{ij}$ = *W*${}_{ji}$. If Wii is output to 0, then the neuron has no connection with itself. It is called the Hopfield network without self-feedback. If *W*${}_{ii}$ output is not 0 it means a Hopfield neural network with self-feedback structure. However, considering the stability of the network, it should avoid using networks with self-feedback.

**Figure 2:**
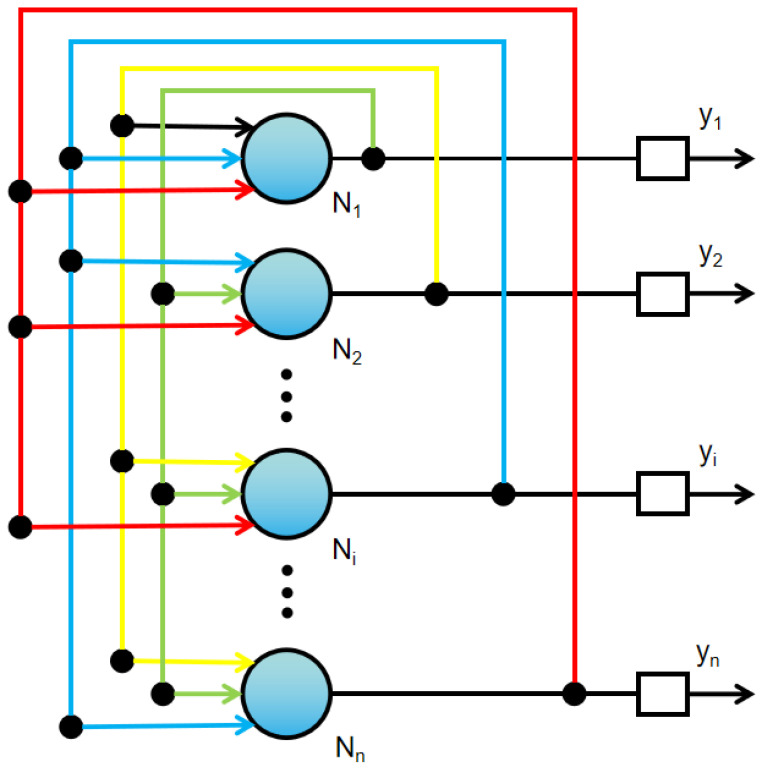
Discrete Hopfield neural network structure.

The fully connected network structure of the DHNN is shown in Figure 2. The output of the neuron *N* is used as the input of other neurons. Then, other neurons will also return the output to themselves. In the Figure 2, *y* represents the neuron output at the next moment. Each neuron of DHNN only takes discrete binary values of 0 or 1. *W*${}_{ij}$ determines the weight between neuron *i* and neuron *j*. Neurons have current state *u*${}_{i}$ and output *v*${}_{i}$ [27]. The *u*${}_{i}$ can be continuous value in the processing, but *v_i_* is binary value in discrete models. The relationship between neuron state and output is as follows, which is the discrete Hopfield neural network evolution Equations (1) and (2).

(1) $$u_{i}\left(t+1\right)=\sum_{j=1}^{n}W_{\mathrm{ij}}v{}_{j}\left(t\right)+I{}_{i}$$

(2) $$v_{i}\left(t+1\right)=f\left(u_{i}\right)=\left\{\begin{array}{ccc} 1 & \mathrm{if} & u_{i}>0\\ 0 & \mathrm{if} & u_{i}\leq 0 \end{array}\right.$$

where *I*${}_{i}$ is the continuous external input of neuron *i*, and *f* () is the activation function of the network. When used for associative memory applications, the weights remain stable after the network training is completed. At this point, the network only two variable parameters, which are updated state and the output of the neurons. Due to the random updating of the neurons, the model is discrete and random on the network. When the network is updated, if the weight matrix is symmetric to the non-negative diagonal, the energy function can achieve minimized value until the system converges to a stable state. When DHNN is designing the connection weight, the stable state of the system is proposed. At this point, the available weight matrix *W* can be obtained through the learned memory of the network. After the learning, the associative network can be achieved by the network weight calculation of output. For trained M models, the DHNN can learn depending on Hebb rules.

The association memory process of the Hopfield neural network indicates that patterns memorized in the neural network are stored on the weight matrix, so the training and learning processes of the weight matrix on the Hopfield neural network becomes particularly important. Hopfield neural networks usually use Hebb learning rules to complete the weight training of neurons. This learning method, proposed by Hebb [28] is the earliest and most famous training algorithm; it still plays an important role in various neural network models. The Hebb rule assumes that when two neurons are excited at the same time, the strength of the connection between them should be strengthened. This rule is consistent with the biological theory of conditioned reflex, which was later confirmed by the neurocyte theory. The Hebb algorithm in the Hopfield neural network can be simply described as one processing node receiving an input excitation signal from another processing node, and if both are at a high excitation level, the weight between processing nodes should be enhanced. Mathematically, the connection weight of the two nodes will be changed according to the product of the two nodes of excitation levels and can be described as:

Hebb Learning Rule on the Hopfield Neural Network [29] by Equation (3).

(3) $$\Delta W_{\mathrm{ij}}=W_{\mathrm{ij}}\left(n+1\right)-W_{\mathrm{ij}}\left(n\right)=\eta Y_{i}X_{j}$$

where *W*${}_{ij}$ (*n*) represents the connection weight from node *j* to node *i* before the *n* + *1* adjustment; *W*${}_{ij}$ (*n* + *1*) is the node *n* to node *i* after the *n* + *1* adjustment of connection weight; η is the learning rate parameter; *X*${}_{j}$ is the output of node *j* and input to node *i*; *Y*${}_{i}$ is the output of node *i*. The objective of using a Hopfield neural network with Hebb Learning method is for a set of *q* different input samples *P*${}_{nxq}$ = [*p${}^{1}$, p${}^{2}$, … p${}^{q}$*]. It is to adjusts the weight matrix *W* to reach a group of input samples *p*${}^{k}$, *k*  =  1,  2,  3,  …, *q* as the initial value of the network, the system can converge to the respective input sample vectors.

## 3. Hardware Design

**Figure 3:**
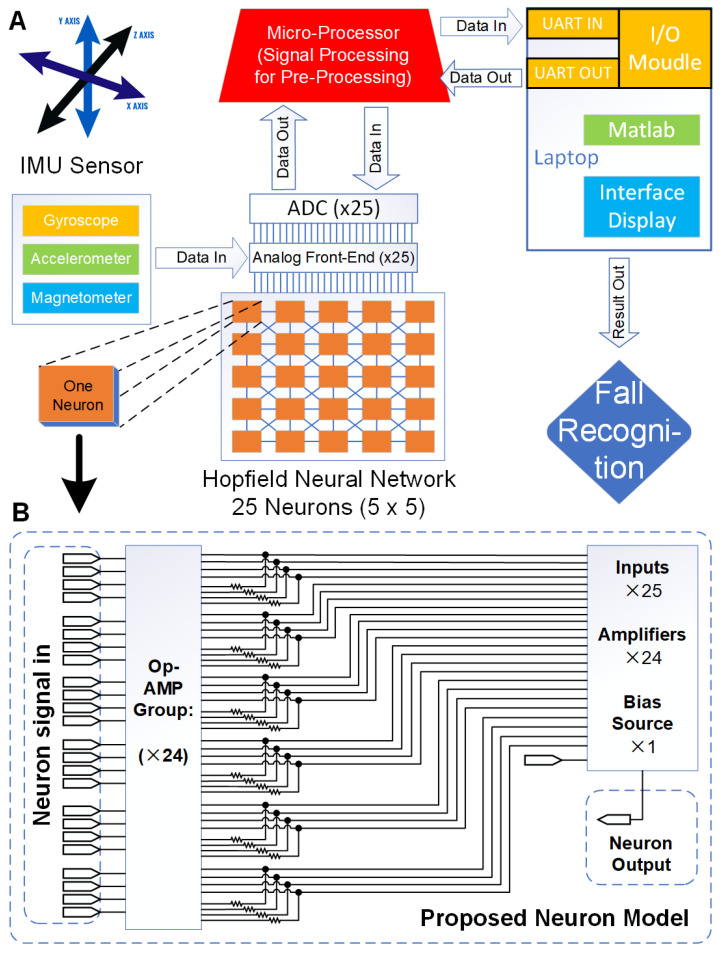
Hardware architecture for system blocks.

A specific application based on Neuromorphic computing hardware to implement neural network model uses the physical unit simulating the neurons process, and the communication units between the neurons to simulate connections of the neural network. Among them, each neuron and each connection has corresponded to the physical design. The advantage of the hardware implementation is processing speed and easiness of execution to satisfy the real-time requirement. In addition, appropriate hardware design can further, reduce energy consumption on the system. Figure 3A shows that the overall fall recognition system and data stream processing workflow. It gives a schematic diagram of neuromorphic computing hardware. We completed the Hopfield neural network algorithm based on the design of 25 neuron modules construction, and its dependence on the associative memory function to finally achieve the fall recognition result by the sensor signal.

The Hopfield neural network hardware design uses analog circuits composed of resistors, operational amplifiers and other components to describe the neurons. The objective function is converted into the energy function of the neural network, and the model of the corresponding pattern is memorized by the equilibrium point of the network energy function. Hopfield network as a recurrent neural network that includes feedback connection from output to input. The designed Hopfield neural network model is shown as in Figure 3B.

### Neuron’s Hardware Design

The state of neurons expresses the result of the neuromorphic processing information. Neurons are the smallest unit of information processing on the neural network. These neurons are connected by a rule to form a physiological neuron network system to the brain. Among them, the strength of the connection between each neuron changes adaptively according to the excitation degree of the external signal. Each neuron presents a state of excitation or inhibition state with the combined magnitude of the received multiple excitation signals. Excitation state refers to the change of neurons from relative rest to relative activity, while inhibition state refers to the change of neurons from relative activity to relative rest. As a result, there are two kinds of connections between the transmission of information between neurons. Positive connections stimulate each other, and negative connections inhibit each other.

The structure of the discrete Hopfield neural network is a single-layer full feedback network architecture, and in this work, we used a total of 25 neurons as 5 × 5 network. Each neuron through the connection weight to receives information from the output of all other neurons. The purpose is to make the output of any neuron controlled by all other neurons, which means each neuron can restrict each other. The essence of associative memory on the discrete Hopfield neural network is that the memory sample to be stored and represented by a vector. After inputting the memory sample, the neural network weight is stable on the memory sample after evolution. When the input of the neural network is a nonlinear memory sample, the output of the network should be either stable in the memory sample; or it is stable in the nonlinear sample. Therefore, the output of the neural network should be stable regardless of the external input.

The dynamic differential system of biological neurons on the neural network can be simulated by the operational amplifier to achieve learning and associate function such as the biological brain. Therefore, in the above Figure 3, each group of operational amplifiers and their associated resistors constitute a model for representing a neuron function. Each neuron has two sets of inputs, one is a constant bias source signal, and the other is a positive or negative feedback connection from the output of other operational amplifiers (other neurons).

**Figure 4:**
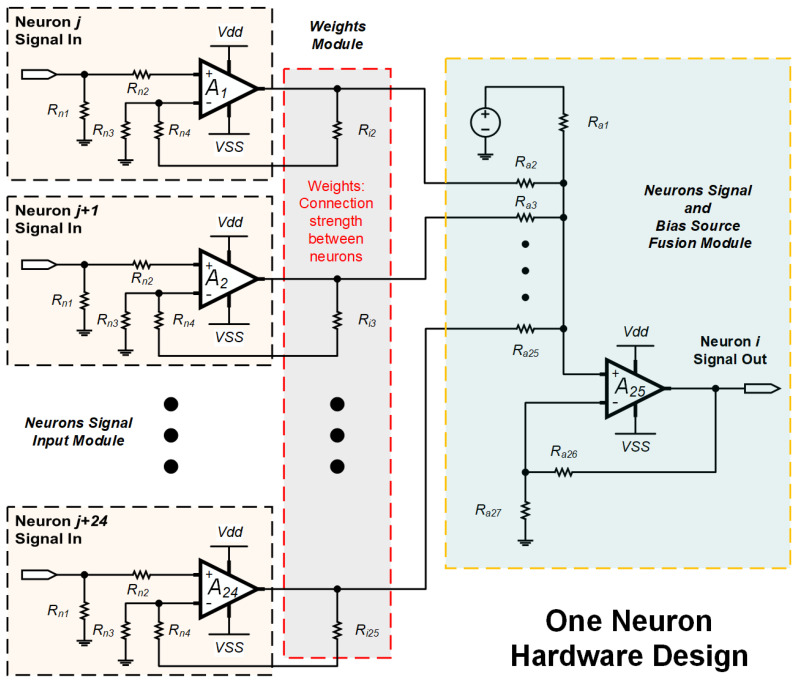
The Hopfield neural network of one Neuron circuit design.

Figure 4 shows the circuit design for one of the neurons, which is a general neuron module for the neuromorphic computing of Hopfield neural network. Based on the neurons connect concept, it includes Neurons signal input module, Weight module and Source fusion module. The Neurons signal input module is to link the other neurons output, so that the work is complete in 24 modules to connect with other 24 neurons (This Hopfield neural network designed 25 neurons, and each neuron link to each other neurons). The Weight module is designed to neurons connection strength representation, which depends on the resistors to control the amplifier gain. The corresponding parameters are calculated by Hebb learning when the neural network training. The Source fusion module as additional computing to fuse the bias source and 24 neurons input. Meanwhile, this module is a data input interface on the Hopfield neural network hardware. The 25 neurons of Hopfield neural network can input 5 × 5 matrix data size to processing, which is each neuron to match one of matrix dot (Arranged dot by rows to input).

**Figure 5:**
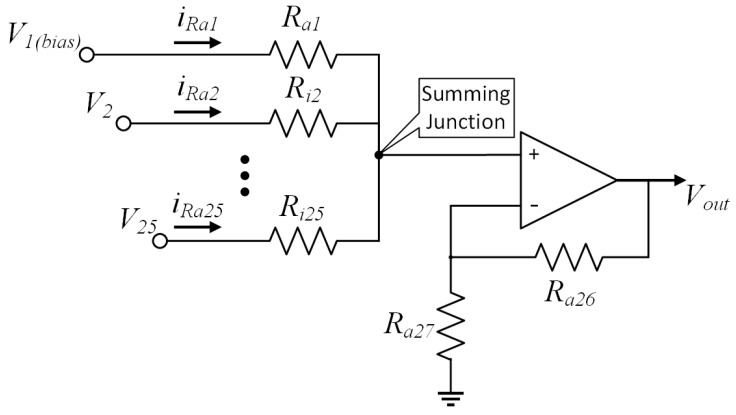
The equivalent circuit of Hopfield neural network.

In the equivalent circuit design of one neuron in Figure 5, the internal membrane potential of the neuron *i* is *V*${}_{i}$ (*i* = 1, 2, …, *n* without self-position, it is a bias source input point), the transmission resistance of the cell membrane is *R*${}_{i}$, the output potential of the neuron is *V*${}_{out}$, the external input current is *V*${}_{bias}$. The resistance *R*${}_{i}$ (*i* = 1, 2, …, *n*) to simulate the synaptic properties between the *i* and *j* neurons. At this point, the function of the amplifier as a summing circuit is to simulate the activation function of the neural network, and the parallel resistance can adjust the connection strength between the neurons. In the equivalent circuit calculation of a neuron, which is based on Kirchhoff’s Current Law (KCL), in a lumped circuit, all the node at any moment, it is the algebraic sum on the identity of the branch currents for all outgoing nodes is zero. The inflow current and outflow current at the input node of the amplifier is maintained balance to achieves neuron voltage signal output. The rest 24 neurons can be done in the same manner that is following by corresponding connection strength. Meanwhile, each operational amplifier simulates the nonlinear characteristics between the input and output of the neuron through Equation (4).

(4) $$V_{out}\left(t\right)=f_{i}\left(\sum_{i=1}^{n}V_{i}\left(t\right)\right)$$

**Figure 6:**
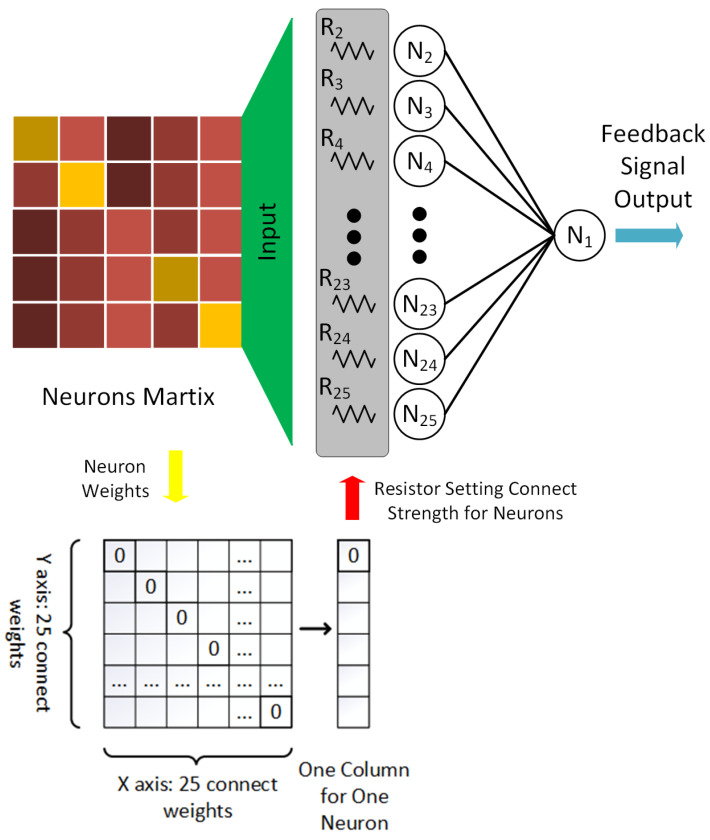
The Hopfield neural network of Neuron Matrix and Weight.

Among them, *f*${}_{i}$ represents the transfer function of the neuron *i*, and defines *W* = *R*${}_{ij}$ (*i, j* = 1, 2, …, *n*) as the weight coefficient matrix of the neural network. The weight is derived from the matrix value obtained by training on the Hebb algorithm. Figure 6 shows the details about the relationships between the weight and matrix.

## 4. Evaluation and Results

In this study, we used Cadence PSpice for circuit design, integrated with MATLAB Simulink to complete the system construction and validation. Meanwhile, the IMU sensors captured 5 classes of human activities as a dataset to achieve data classification for fall detection, which includes Fall, Sit down, Stand up, Under and Walk sensor signal. The neuromorphic computing of Hopfield neural network algorithm was used to classify the human fall activity data as a positive sample and other activities as negative samples to complete fall detection.

### 4.1. Data Preprocessing for Feature Extraction

**Figure 7:**
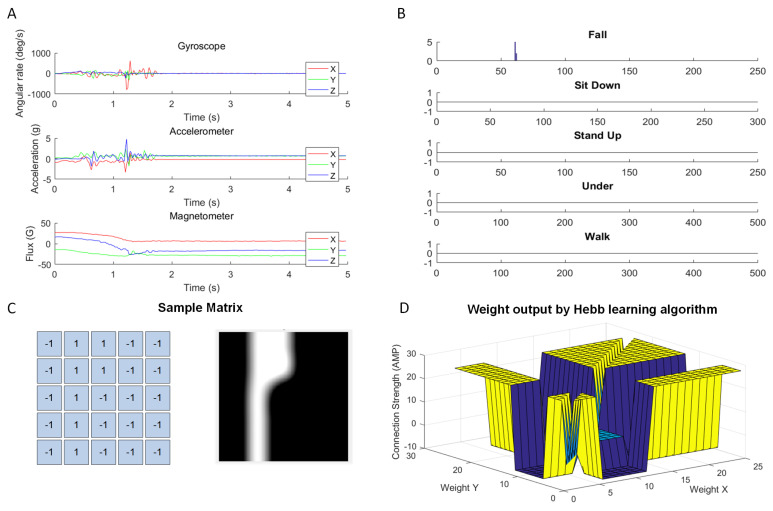
The Sensor data converted into Hopfield neural network of sample and weight, (**A**) Raw IMU sensor signal output, (**B**) Sensor data feature extraction and binary conversion, (**C**) Fall activity sample matrix of feature map, (**D**) Hebb learning algorithm output the Hopfield neural network weight.

First, the matrix sample template of human fall activity is achieved by feature extraction computing that is based on data preprocessing for IMU sensor data. There are 9 axis signals acquired by the IMU Sensors, which is sampling frequency is 50 Hz and capture 5 classes activities for 5–10 s. Figure 7A shows the raw IMU signal output to the Fall activity. The project uses a construction matrix to fuse 9-axis sensor data, and then depend on threshold selection to identify human fall activity. The processing results are shown in Figure 7B. It shows the output of the human fall signal in the data has a different value with other classes human activities. To adjust Hopfield Neurons circuits design, a sample template of human fall activity is extracted from the fused computing results. Due to the symmetry characteristics of the Hopfield neural network, it flipped the fall signal output to 5 × 5 feature template to facilitate the visualization of the weight output. The 5 × 5 Fall sample template as shown in Figure 7C, which is matched with 25 neurons matrix on the circuits design. For the hardware design, the Neuron Weight is calculated by the Hebb Learning algorithm. The 5 × 5 Fall activity feature matrix is applied as input to the Hebb Learning algorithm to train with Hopfield neural network architecture of 25 neurons. The trained Weight output is shown in Figure 7D, which is shown the neuron connection relationship on the neural network architecture. Finally, setting the weight value into the Hopfield neural network is taken as circuit parameters to complete hardware design. Algorithm 1 describes the main steps of the sensor data feature extraction and binary conversion, where the first step involves calculating the 9 axes of three sensors data, which is shown in Figure 7A. After the threshold value processing, we achieve a 5 ∗ N binary matrix for which the results are shown in Figure 7B. The suitable threshold values were obtained through iterative trial and error steps, so that the fall activity with other data can be accurately classified. Finally, a sliding 5 × 5 window box decides which 5 × 5 feature map for a fall activity is captured, and it is illustrated in Figure 7C.
**Algorithm 1** Sensor data feature extraction and binary conversion.1:**Initialize matrix M(s, s) to full zero matrices.**2:**Load three Sensors data:**3:[Gx, Gy, Gz] = Gyroscope Sensor data Matrix [:1, 2, 3]4:[Ax, Ay, Az] = Accelerometor Sensor data Matrix [:1, 2, 3]5:[Mx, My, Mz] = Magnetometor Sensor data Matrix [:1, 2, 3]**Require:**6:M${}_{1}$(n) = (abs(Gx) + 1) ∗ (abs(Gy)) + 1) ∗ (abs(Gz)) + 1);7:M${}_{2}$(n) = (abs(Ax)) + 1) ∗ (abs(Ay)) + 1) ∗ (abs(Az)) + 1);8:M${}_{3}$(n) = (abs(Mx)) + 1) ∗ (abs(My)) + 1) ∗ (abs(Mz)) + 1);9:M${}_{4}$(n) = (1/2) ∗ (Ax ∗ My ∗ Gz − Gx ∗ My ∗ Az)10:M${}_{5}$(n) = (1/2) ∗ (Gx ∗ Ay + Ax ∗ My + Mx ∗ Gy − Gx ∗ My − Ax ∗ Gy − Mx ∗ Ay)11:**Binarization and Feature Extraction:**12:**Threshold value**: t = [16, 24, 32, 40, 48];13:**for** i = 0:4 **do**14:out[n][i] = (M > t[i]) ? 1 : − 1;15:Update the Matrix M with the Binarization line by line.16:return **Matrix M(5:N)**;17:Sliding window box **do** M = M(starti:starti + 4, :)18:**end**19:return **Matrix M(5:5)**;

### 4.2. Comparison with State-of-the-Art Machine-Learning Algorithm and Discussion

In the test, saving the Hopfield Neuron’s output feedback signal as a binary matrix (high voltage level represents Binary value 1, and low voltage level represents Binary value −1), and then using the cosine distance method [30] the feedback matrix is computed to achieve the confidence level and accuracy of the classification. Finally, following the accuracy calculation (True positive (TP) + True negative(TN))/(True positive (TP) + False positive(FP) + True negative(TN) + False negative(FN)), the fall activity classification result that has been achieved is 88.9% on the hardware-based design. Table 1 shows comparison results for different algorithms and their respective platform accuracy. Such as Li et al. [31] worked on the same dataset, and they used traditional machine-learning algorithm on the MATLAB platform. They have deployed Support Vector Machine (SVM) and Artificial Neural Network (ANN) to classify sensor and radar data for fall detection. Meanwhile, there are many machine-learning algorithms on low-power platforms to achieve fall detection. Based on the ZYNQ hardware of the FPGA platform, Nguyen [32] implements the Gaussian Mixture Model by digital design, and Garg et al. [33] deploys the Deep Neural Network (DNN) algorithm through the ARM processor to calculate the sensor data of the fall activity. However, through comparing accuracy, our implementation is more accurate than their classification. In addition, comparing training dataset requirements, it can be found that machine learning and deep learning rely on a large number of samples to learn features. The learning strategy of neuromorphic computing is more friendly with limited sample datasets. The model template of the fall sensor signal is obtained by preprocessing the fall feature extraction. Depending on the Hebb learning method, the associative memory of the sample is realized on the Hopfield neural network. In this way, only one sample learning is requested on the training and then the fall activity recognition weight is achieved for the Hopfield neural network.

Furthermore, our proposed project also compares the software-based (MATLAB) output with Hardware-based approaches (PSpice-Matlab Simulink co-simulation results). For the hardware circuit design, slight differences can occur between analog signal transmission, which is different from the software environment and its respective ideal calculation. However, depending on the associative memory function for the Hopfield neural network, the analog noise has been reduced. Compared with conventional machine-learning algorithms, better outcomes are obtained with neuromorphic computation after careful extraction of the function and configuration of the model models. However, it prevents the cycle of internal loss and noise effects on the analog circuit, relying on the binary approximation to create the neuromorphic computing algorithm. For the complexity and precision requirements of analog and digital conversion equipment, neuromorphic computing is closely integrated with the analog circuit’s performance, which enables systems to reduce the impact on the accuracy.

## 5. Conclusions

In this paper, a neuromorphic computing hardware design both in the software and hardware domains with sensor data fusion method is proposed. It is co-designed especially for health care applications. where we use neural network hardware to achieve low-power and real-time operation for multiple sensors data in an embedded system. The neurons stimulated by amplifiers, resistors and other components, which are used for processing inhibition and activation signals on the neurons. The neural network is then constructed by combining neurons. By designing the front-end feature extraction algorithm, the multiple sensor data fusion results are achieved, which are then passed to the hardware-based neural network to finally generate the recognition result. This proposed hardware and software co-design solution can be used for smart wearable devices in healthcare applications and can perform local real-time data processing, which does not require the additional cloud computing processes for data interaction with sensors. The low-power and real-time hardware can simulate neuromorphic computing with the help of which machine learning can be applied for data processing. Results show that through simulations, the hardware-based neuromorphic computing of the fall data reached an accuracy of 88.9% which is at least 1.6% greater than the state-of-the-art algorithms, such as SVM, ANN and DNN. The neuromorphic computing hardware design proposed in this paper is a simple, fast and reliable solution. In our design, a straightforward circuit design is used to control the weight setting from the Hopfield neural network which is based on the resistance to vary the amplification factor, which essentially maximizes the reusability and flexibility of the circuit design. The proposed design can reduce the design time and help avoid separate debugging processes of the amplifier to easily adjust the weight setting. We believe that the design has the potential to exploit the neuromorphic computing framework for data fusion in healthcare applications.

## Figures and Tables

**Table 1:** Comparison table with different methods and environments.

Project	Algorithm	Environment	Training Dataset	Accuracy
Our Work (Hardware -based)	Hopfield Neuron design on analog circuit	PSpice (Analog Design) + Matlab Simulink (Only Data Transit) Co-Simulation	1 Sensor sample to generate a standard pattern for each activity	88.9%
Our Work (Software -based)	Hopfield Neural Network	Matlab (Code)		94.4%
Li et al. [31]	Support Vector Machine (SVM)	Matlab (Code)	Using a 70% Sensor dataset as training data (20 volunteers ∗ 3 repetitions ∗ 70% = 42 training samples for each activity)	79.83%
	Artificial Neural Network (ANN)		Using a 70% Radar dataset as training data (20 volunteers ∗ 3 repetitions ∗ 70% = 42 training samples for each activity)	85.53%
Nguyen [32]	Gaussian Mixture Model (GMM-HMM)	FPGA (Digital Design)	DUT-HBU dataset is used and all video data are compressed in avi format and captured by a single camera in a small room with the changeable conditions	87.3%
Garg et al. [33]	Deep Neural Network	ARM (Tensorflow)	A sample of data points from 2.5 s before and after the spike, making a total of 200 ∗ 5 = 1000 discrete samples [33]	86.2%
